# V2X-Communication-Aided Autonomous Driving: System Design and Experimental Validation

**DOI:** 10.3390/s20102903

**Published:** 2020-05-20

**Authors:** Chanyoung Jung, Daegyu Lee, Seungwook Lee, David Hyunchul Shim

**Affiliations:** 1 Department Electrical Engineering, Korea Advanced Institute of Science and Technology (KAIST), 291 Daehak-ro, Yuseong-gu, Daejeon 305-338, Korea; lee.dk@kaist.ac.kr (D.L.); hcshim@kaist.ac.kr (D.H.S.); 2 Robotics Program, Korea Advanced Institute of Science and Technology (KAIST), 291 Daehak-ro, Yuseong-gu, Daejeon 305-338, Korea; seungwook1024@kaist.ac.kr

**Keywords:** autonomous driving system, V2X communication, intelligent transportation system, perception, planning, control

## Abstract

In recent years, research concerning autonomous driving has gained momentum to enhance road safety and traffic efficiency. Relevant concepts are being applied to the fields of perception, planning, and control of automated vehicles to leverage the advantages offered by the vehicle-to-everything (V2X) communication technology. This paper presents a V2X communication-aided autonomous driving system for vehicles. It is comprised of three subsystems: beyond line-of-sight (BLOS) perception, extended planning, and control. Specifically, the BLOS perception subsystem facilitates unlimited LOS environmental perception through data fusion between local perception using on-board sensors and communication perception via V2X. In the extended planning subsystem, various algorithms are presented regarding the route, velocity, and behavior planning to reflect real-time traffic information obtained utilizing V2X communication. To verify the results, the proposed system was integrated into a full-scale vehicle that participated in the 2019 Hyundai Autonomous Vehicle Competition held in K-city with the V2X infrastructure. Using the proposed system, the authors demonstrated successful completion of all assigned real-life-based missions, including emergency braking caused by a jaywalker, detouring around a construction site ahead, complying with traffic signals, collision avoidance, and yielding the ego-lane for an emergency vehicle. The findings of this study demonstrated the possibility of several potential applications of V2X communication with regard to autonomous driving systems.

## 1. Introduction

The last few decades have seen tremendous interest in the development of autonomous vehicles for road safety, driver convenience, and traffic efficiency, from both academia and industry. The Society of Automotive Engineers (SAE) has defined standards for autonomous vehicles divided into six levels according to driving automation levels, from zero automation to complete automation. Autonomous vehicle systems above Level 4 are mainly divided into three subsystems: perception, planning, and control. The perception subsystem extracts meaningful information from the sensing data using on-board sensors such as cameras, LiDARs, and radars. In the planning subsystem, functions such as action prediction, path planning, and obstacle avoidance are combined to generate an effective plan in a real-time (RT) manner. Finally, the controlling subsystem governs the longitudinal and lateral motions of the vehicle based on this plan. Although various methods have been proposed to enhance the robustness of the perception, planning, and control subsystems, the limited perception range of sensors is a natural limitation that cannot be overcome; this also has a considerable negative impact on the performance of subsequent planning and control subsystems. With the emergence of vehicle-to-everything (V2X) communication [1], autonomous vehicles have the ability to identify blind intersections, as well as drive under bad weather conditions. V2X also enables vehicles to obtain comprehensive information, which the sensor alone cannot record (e.g., traffic status, detailed states of neighboring vehicles, and information regarding construction sites that lay ahead). Based on the aforementioned characteristics, V2X shows significant potential for ensuring road safety, fuel efficiency, and manageable traffic flows [2,3,4,5]. Cooperative adaptive cruise control (CACC) or platooning by grouping two or more consecutive automated vehicles traveling along with a lead vehicle is one of the most well-known applications where V2X has been used for intelligent transportation. Typically, CACC employs a spacing strategy to maintain the required distance between vehicles using state information of the lead vehicle delivered via V2V communication. Thus, the target speed of the following vehicle (high-level command) can be determined by the lead vehicle for ensuring fuel efficiency and road safety. Researchers [6] have developed a low-level controller for CACC focusing on platoon-level automation considering the uncertainties of vehicle dynamics. The effectiveness of the said controller has been experimentally demonstrated via connected cruise control considering the situation in a partially automated traffic environment, which is a very realistic approach [7]. Additionally, researchers [8] have experimentally validated the impact of connected cruise control with various connectivity topologies, thereby demonstrating that the connected automated vehicles can attenuate velocity perturbations in human-dominated traffic.

Similar to the studies mentioned above, most extant studies employing V2X for intelligent transportation utilized V2X systems for performing one task in automated vehicles, such as car following or lane changing. However, in highly automated vehicles (Level 4 and above) that can reach their destination without driver intervention, these systems must be able to perform a variety of functions with a well-organized system architecture and cope with various situations in the road environment. In addition, the many studies related to V2X systems have analyzed the performance of these systems or their algorithms in simulation environments. The main reason for this is that V2X technology has emerged recently, and there are not many testbeds available with the V2X infrastructure yet. However, for autonomous driving in real-world environments, performance verification through simulations is not enough because various real-world problems such as communication delays, traffic situations, and limited computing resources must be considered.

Therefore, in this paper, we propose a V2X-aided autonomous driving system for highly automated vehicles; we verified its performance through real-world experiments. The proposed system is composed of a V2X system playing a supplementary role with various core algorithms for autonomous driving functions (navigation, control, planning, etc.). That is, the proposed system does not depend on the existence of V2X infrastructure, and it is configured to enable autonomous driving in situations where V2X communication is impossible for some reason. Our proposed autonomous driving system consists of three subsystems: beyond line-of-sight (BLOS) perception, extended planning, and controlling. To overcome the natural drawback of the on-board sensor, i.e., its limited line-of-sight (LOS), the BLOS perception system performs data fusion between the information received through V2X. The on-board sensor recognizes the local perception results. This broadens the perception range of autonomous vehicles and enables reliable perception in occlusion environments such as intersections. In the extended planning subsystem, the global path planning algorithm considers the RT traffic status received from the V2X system and the local path planning algorithm to avoid collision, and velocity and behavior planning algorithms are executed. Based on the planning results, the controlling subsystem of the autonomous vehicle performs lateral and longitudinal control of the vehicle. We integrated our autonomous driving system into a full-scale vehicle platform (Hyundai i30) and participated in the 2019 Hyundai Autonomous Vehicle Competition (AVC) to validate its performance. The competition was held in K-city, an unpopulated city for autonomous vehicle testing based on V2X networks, and consisted of various missions such as pedestrian crossing, traffic light observation, and accident avoidance. We won the third place with all missions completed.

The remainder of this paper is organized as follows. Section 2 provides a brief introduction to V2X concepts and the messages transmitted in K-city. The full-scale autonomous vehicle platform incorporating the proposed system is discussed in Section 3. In Section 4, we introduce the algorithms and key features of the BLOS perception, extended planning, and control subsystems that make up the V2X-aided autonomous driving system. In Section 5, experimental results for performance validation are discussed. Finally, we conclude the paper with discussions on our approach and future research directions to further improve our autonomous driving system.

## 2. Introduction of V2X in K-City, the Autonomous Vehicle Test Bed

V2X is a communication technology that exchanges traffic information with other vehicles and road infrastructure through wired/wireless networks while driving. One example is vehicle-to-vehicle communication (V2V), which allows vehicles to communicate with one another. V2X is comprised of V2V, vehicle-to-infrastructure (V2I), vehicle-to-pedestrian (V2P), and vehicle-to-nomadic device (V2N) communications, thereby including all forms of communication technology applicable to vehicles on the road [9].

Currently, there exist two major V2X technologies: LTE-V2X and dedicated short-range communication (DSRC) [10,11]. LTE-V2X is a vehicular wireless communication technology based on the 4G cellular service that facilitates communication with other vehicles and mobile devices with the aim to support V2X and non-safety applications. DSRC, also known as wireless access in vehicular environment (WAVE), is another major V2X communication technology. The WAVE architecture includes IEEE P1609.1 (application), IEEE P1609.2 (security), IEEE P1609.3 (network), IEEE P1609.4 (upper MAC), and IEEE 802.11p (lower MAC and physical) layers [12]. The J2735standard specifies a message set along with its data frames, as well as data elements specifically for use in applications intended to utilize the 5.9-GHz DSRC communication systems [13]. An open-source ASN.1 compiler was used to decode these J2735 messages.

In this study, we designed an autonomous driving system aided by DSRC V2X. Our system was tested in K-city, an unpopulated city for autonomous vehicle testing built with V2X infrastructures. As described below, J2735 messages broadcast in K-city are comprised of four standard message types: basic safety message (BSM), traveler information message (TIM), signal phase and timing message (SPAT), and MAPmessage.

- Basic safety message (BSM): The BSM is a representative message type of V2V communication. It contains the core data elements such as vehicle size, position, heading, acceleration, and brake system status. In addition, it contains such information as vehicle type, description, and identification. It is transmitted at approximately 10 Hz.
- Traveler information message (TIM): The TIM is used to convey information regarding different traffic conditions. It is the means to inform the public about both incidents (traffic accidents) and pre-planned roadwork events such as road construction. It is transmitted at approximately 1 Hz.
- MAP Message: The MAP message is used to provide geometric data on intersections and roadway lanes. This message is used to number and describe lane-level details for each lane at an intersection. It is transmitted at approximately 1 Hz.
- Signal phase and timing message (SPAT): The SPAT message is used to provide signal and phase-timing data for one or more intersections. All SPAT messages are linked to MAP messages to convey road details and link signal-controller steps to the correct set of lanes. It is transmitted at approximately 1 Hz.

## 3. Full-Scale Autonomous Vehicle Platform, Eurecar

**Figure 1:**
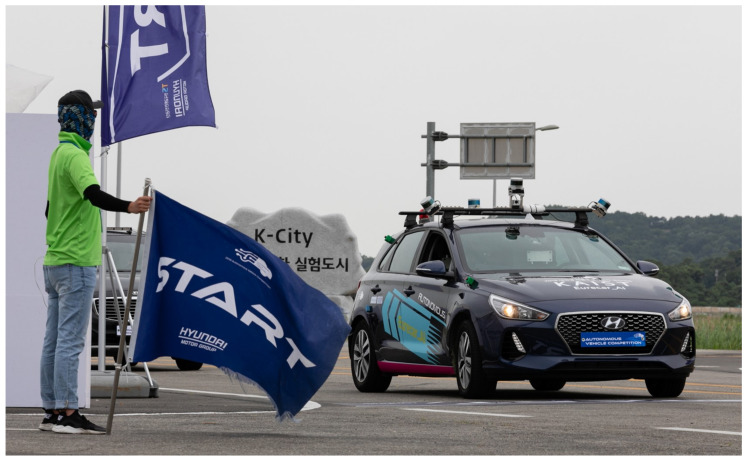
Full-scale autonomous vehicle platform, Eurecar, in K-city.

Figure 1 shows our full-scale autonomous vehicle platform, Eurecar, built by retrofitting a Hyundai i30 vehicle model. The vehicle was equipped with an additional steering motor for lateral control and wire actuators for the accelerator and brake pedal. For development purposes, emergency stop switches were configured in and out of the vehicle. For precise inertial navigation, Ublox M8P GPS and real-time kinematic (RTK) units, Microstrain’s 3DM-GX4-25 inertial measurement unit, and a wheel odometer received from CANinformation were fused using with an extended Kalman filter. For the on-board sensory system, four multichannel LiDARs and a front-facing vision sensor were installed. The LiDAR sensors were mounted on the vehicle roof and extrinsically calibrated based on their position and orientation. Three LiDARs mounted near the front axle of the vehicle were extrinsically calibrated with the front-facing camera. For wave-based V2X communication, a receiving antenna and an on-board unit (OBU) were installed on the roof.

Eurecar’s computing system consists of four computers, depending on the operating system (OS) and purpose. NVIDIA’s automotive-level AI computer, DRIVE PX2, was responsible for driving environment awareness using vision sensors, and HP Omen with Linux was responsible for camera–LiDAR fusion and LiDAR-based driving environment recognition, receiving data from the V2XOBU and various planning algorithms. The other two computing devices were for controlling the vehicle. Among them, the NI Compactrio was responsible for low-level control of the motor involving lateral and longitudinal control with an RT OS. The last one was a high-level mission computer. It monitored all the other computers and planned the behavior of the autonomous vehicle. All the computers communicated over a gigabit Ethernet link with Robot OS (ROS) and lightweight communications and a marshaling communication protocol.

## 4. V2X-Aided Autonomous Driving System Architecture

**Figure 2:**
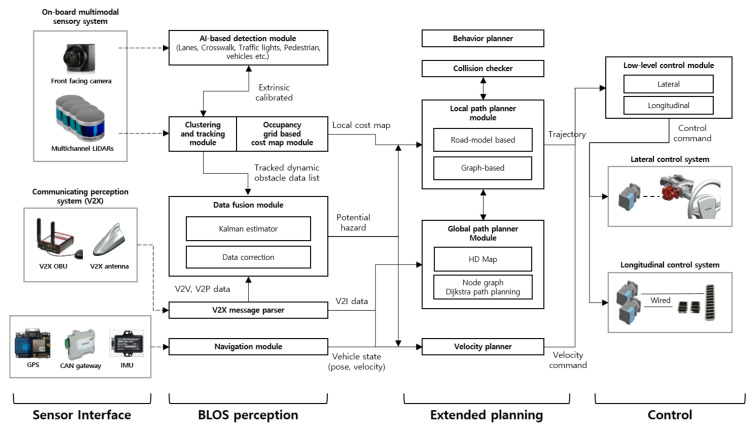
Overall architecture of the Eurecar autonomous vehicle system. BLOS, beyond LOS.

Figure 2 illustrates the overall system architecture, which consisted of three major subsystems, i.e., BLOS perception, extended planning, and control. The following subsections describe the key features of each subsystem and introduce the core algorithms.

### 4.1. BLOS Perception Subsystem

Autonomous vehicles require reliable perception and understanding of the environment to perform accurately. The performance of the perception system will considerably affect the overall ability and robustness of the system. Recently, autonomous driving perception systems have evolved tremendously by incorporating deep learning techniques with advances in sensing and computing technologies [14,15,16,17]. Despite these significant advances, the limited LOS of on-board sensors is insurmountable, and the performance of the perception subsystem is easily affected by environmental factors such as weather and road conditions. V2X communication is the information exchange between a vehicle and various elements of an intelligent transportation system, including other vehicles, pedestrians, and transport infrastructure; it is seen as an alternative that overcomes the drawback of the limited LOS of on-board sensors, and it is proven that V2X can improve road safety. However, to realize all the advantages of V2X in autonomous vehicles above Level 4, important challenges remain to be overcome; these challenges include the need for data that are more precise, lower transmission delay, and traffic loss handling during communication [18,19]. Table 1 lists the strengths and weaknesses of local perception with on-board sensors and communication perception via V2X communication mentioned above.

Based on the features of these two approaches as summarized in Table 1, we designed and integrated the BLOS perception system into Eurecar, which combined local perception using a multimodal sensory system and communicated perception with V2X. It did not rely on either just on-board sensing or V2X and was designed to exploit their strengths and mutually compensate their weaknesses via a data fusion algorithm. The following are the details about local perception and phases for fusion with V2X.

**Figure 3:**
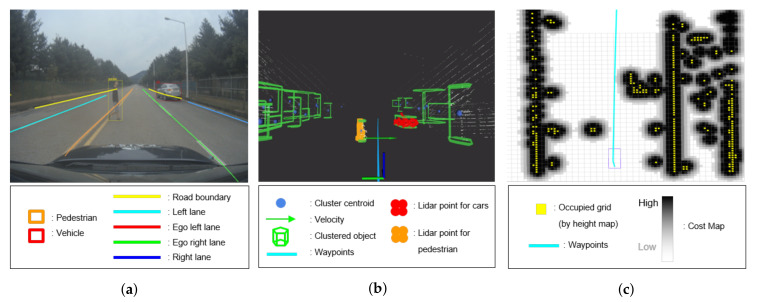
Visualization of local perception result. (**a**) Vision-based multi-class detection result obtained using Drivenet. (**b**) Clustered LiDAR data in the 3D coordinate system using the DBSCAN algorithm. Using vision-based detection result, clusters corresponding to dynamic objects (pedestrian and neighboring vehicles) can be tracked for estimating relative velocity. (**c**) Cost map obtained from occupancy grid information with yellow indicating the grid to be occupied by obstacles. In general, the grid cost increases as the color changes from white to black.

The local perception subsystem aimed to recognize accurately the objects and drivable regions within the LOS using on-board sensors in RT. We realized local perception using cameras and LiDARs, which are typical sensors used in autonomous vehicles. The front-facing camera detected the lanes, neighboring vehicles, pedestrians, traffic lights and signs, and drivable regions in the image (Figure 3a) using Drivenet [20]. Drivenet is a camera-based deep neural network model (nine inception layers and three convolutional layers) designed by NVIDIA for detecting the multi-class and location of the target objects. Running on a DrivePX2 computer, it runs at 22 frames per second. In parallel, the LiDAR point clouds acquired through the four LiDARs mounted on the roof were clustered using the density-based spatial clustering of applications with noise (DBSCAN) algorithm [21]; the relative velocity of the cluster was estimated using the interacting multiple-model unscented Kalman filtering (IMM-UKF) algorithm [22]. The location of the detected object in the image was determined using simplified three-dimensional (3D) spatial information through clustering and the extrinsic matrix between the camera and LiDAR obtained from offline calibration (Figure 3b). Moreover, to recognize the drivable region, an occupancy grid map considering negative and positive obstacles was generated (Figure 3c).

For the BLOS perception system, we performed data fusion with V2X only for dynamic obstacles recognized through local perception. Vehicles and pedestrians are the most representative dynamic obstacles in driving environments; these have a significant influence on the stability of autonomous driving in occlusion environments such as intersections and blind corners. V2V and V2P were used to provide the location and velocity of the vehicles and pedestrians, respectively. The data fusion algorithm (Algorithm 1) consisted of three steps: (1) converting the global position of V2V and V2P into body coordinates based on the current vehicle position and orientation; (2) estimating the position and velocity of the object recognized via on-board sensors; and (3) calculating all the Euclidean distances between the local perception and communicated perception to determine whether they referred to the same object and generating a dynamic obstacle list containing the position, velocity, and orientation.

(1) $$R_{h}=\left\{\begin{array}{cc} 0 & \mathrm{if}\;D_{c}>D_{long}\\ \frac{k_{p}\times max\left(0,proj_{u}v\cdot u\right)}{TTC}+\frac{k_{l}}{\min(\left|u\right|,0.1)}\; & \mathrm{else} \end{array}\right.$$

where: $k_{p}$: gain for longitudinal direction; $k_{l}$: gain for lateral direction; $E_{v}$: velocity of the ego-vehicle; time-to-collision (TTC) = $\frac{D_{c}}{E_{v}}$.


**Algorithm 1:** Data fusion algorithm with local and communicated perception.

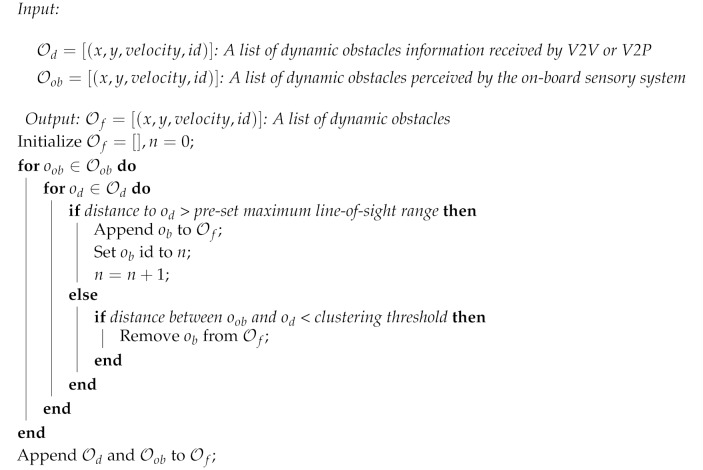




**Figure 4:**
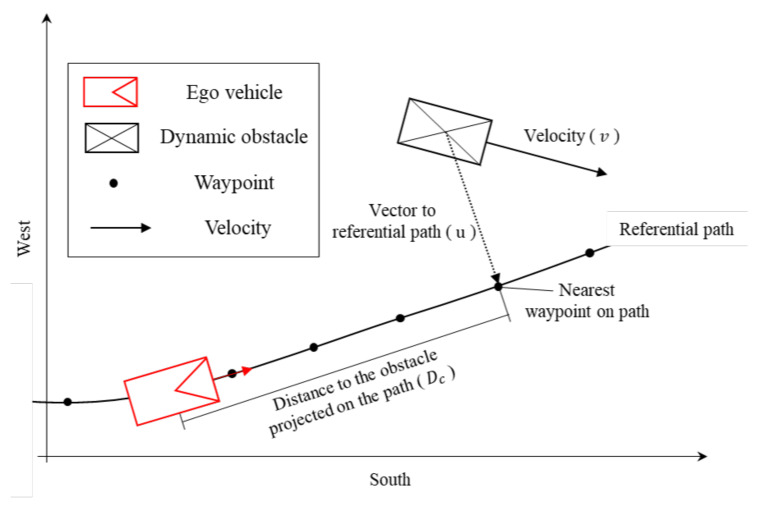
Schematic of potential hazard calculation using dynamic obstacles.

The dynamic obstacles recognized through the BLOS perception system were assigned to the potential hazards calculated using Equation (1). Figure 4 visually depicts the schematic of potential hazard calculation. In Equation (1), $k_{p}$ and $k_{l}$ denote the gain of velocity and the distance to the referential path, respectively. Moreover, $D_{c}$, which represents the distance to the object projected on the path, was compared with a predefined threshold ($D_{long}$), and a potential hazard value was assigned only when the distance was within the range. If the calculated potential hazard was higher than the preset threshold, then Eurecar would slow down to lower the potential hazard.

### 4.2. Extended Planning Subsystem

The planning system of autonomous vehicles can be configured in various ways according to the operating environment, service type, and so on [23,24,25]. This section describes Eurecar’s planning system, which is classified into four parts: global path planner, local path planner, behavior planner, and velocity planner. The global path planner plans the global traveling route to the destination with prior information on the driving environment. Further, the local path planner aims to transform the global plan into a local path considering the dynamic obstacles and vehicle constraints. The velocity planner is responsible for generating a suitable speed command for the vehicle to follow. The behavior planner of the autonomous vehicle focuses on handling on-road traffic rules and safety aspects such as obeying the speed limit and following traffic signs and signals.

#### 4.2.1. Global Path Planning

The global path planning subsystem computes a set of $paths=\{P_{1},P_{2},\ldots ,P_{N}\}$ with the prior map of the driving environment, considering the current route, ego-position, and traffic rules. Each path $P_{j}=\left\{p_{1},p_{2},\ldots ,p_{P}\right\}$ is a sequence of poses $p_{i}=\left(x_{i},y_{i},\theta_{i}\right)$, which are positions and their respective orientations. Eurecar uses a high-definition map (HD map) as the prior map containing the geometric and topological details of road infrastructures such as roadway centerlines, lanes, telephone poles, traffic lights, and signs. We reconstructed the light-weight road network model by extracting the roadway centerline information from the HD map and used it for global path planning.

Reconfiguring the road network model with only the roadway centerline can reduce the computational burden of the autonomous driving system and increase the flexibility of the map. However, as the roadway centerline of the HD map was not provided where lane information is absent, such as at an intersection, we generated the sequence of poses, $P_{j}$, obtained through manual driving as the joint path. This centerline-based road network model consisted of two types of paths (centerline and joint paths) and nodes.

**Figure 5:**
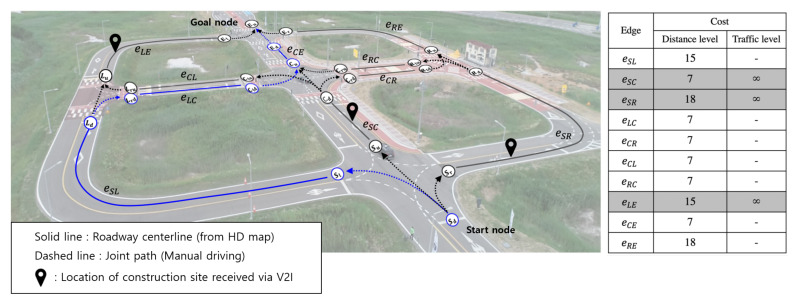
Visualization of centerline-based road network (nodes and edges) in K-city with the route planning result. It was assumed that the global path planner received real-time traffic situation information (construction site locations) through V2I. The roadway centerline from the HD map is represented by the solid line. Dashed lines with arrows indicate the joint path and its direction. White circles indicate the nodes. The table lists the distance-level and traffic-level costs of each edge. By setting the traffic-level cost corresponding to the construction location to infinity, our global path planner planned the minimum-cost path to detour the section.

As shown in Figure 5, we designed both ends of the centerline as nodes and the joint path and centerline as the edges connecting the nodes considering the connection between each node. For global path planning, the conventional road networks were simplified to the shortest path problem in graph theory, which is usually solved using Dijkstra, A-star, and other algorithms [26,27]. However, for autonomous vehicle navigation applications, RT traffic status should be considered and not only the distance of the travel route. We performed cost-minimized global path planning by applying the traffic-level cost related to the RT traffic status received from V2I together with the distance-level cost to the corresponding edges. In this study, we assumed that the traffic status information received through V2I included construction site information and that the section cannot be passed. However, our global planner could be easily extended by additionally configuring the cost function for various traffic conditions such as traffic jams.

#### 4.2.2. Local Path Planning for Dynamic Obstacles

**Figure 6:**
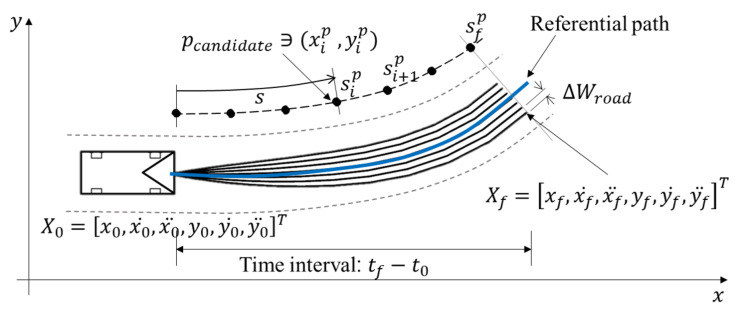
Road-model-based path candidates and global referential path in the Cartesian coordinate system.

The local path planner is responsible for locally replanning the given global path to cope with environmental changes such as encountering dynamic obstacles while autonomous vehicles are moving. Obviously, the local path must be collision-free, while considering the kinematic and dynamic constraints of the vehicle, comfort, and efficiency. We combined both road-model-based and graph-based approaches for local path planning together. The road-model-based approach was used for situations where obstacle avoidance in the lane was required. This approach consisted of two parts: path candidate generation and cost optimal path selection. In the first step, a set of path candidates $P_{candidate}$ are generated using a quintic polynomial fitting algorithm as described in [28]. Geometrically, each path candidate $p_{candidate}$ is generated by connecting the current position ($x_{0},y_{0}$) and sampled endpoint ($x_{f},y_{f}$), which is determined with a different lateral offset $\Delta W_{road}$ (here, we set it to 0.5 m) based on a referential path. To take into account velocity and acceleration limits, each path candidate can be represented with a start state of the vehicle $X_{0}={\left[x_{0},\dot{x_{0}},\ddot{x_{0}},y_{0},\dot{y_{0}},\ddot{y_{0}}\right]}^{T}$ and the state of the endpoint with desired velocity and acceleration $X_{f}={\left[x_{f},\dot{x_{f}},\ddot{x_{f}},y_{f},\dot{y_{f}},\ddot{y_{f}}\right]}^{T}$ with time interval $T:=t_{f}-t_{0}$ as follows:

(2) $$\begin{array}{cc} \; & x\left(t\right)=a_{x0}+a_{x1}t+a_{x2}t^{2}+a_{x3}t^{3}+a_{x4}t^{4}+a_{x5}t^{5}\\ \; & y\left(t\right)=a_{y0}+a_{y1}t+a_{y2}t^{2}+a_{y3}t^{3}+a_{y4}t^{4}+a_{y5}t^{5} \end{array}$$

where $A_{coeff}^{x}={\left[a_{x0},a_{x1},a_{x2},a_{x3},a_{x4},a_{x5}\right]}^{T}$ and $A_{coeff}^{y}={\left[a_{y0},a_{y1},a_{y2},a_{y3},a_{y4},a_{y5}\right]}^{T}$ can be obtained by calculating the inverse matrix of time. To account for the curvature of the referential path as illustrated in Figure 6, the referential path was converted into curvilinear coordinates using a cubic spline. In addition, all the positions of path candidate ($x_{i}^{p},y_{i}^{p}$) can be obtained as follows:

(3) $$\begin{array}{c} x_{i}^{p}=\alpha_{x}{\left(s-s_{i}^{p}\right)}^{3}+\beta_{x}{\left(s-s_{i}^{p}\right)}^{2}+\gamma_{x}\left(s-s_{i}^{p}\right)+\delta_{x}\\ y_{i}^{p}=\alpha_{y}{\left(s-s_{i}^{p}\right)}^{3}+\beta_{y}{\left(s-s_{i}^{p}\right)}^{2}+\gamma_{y}\left(s-s_{i}^{p}\right)+\delta_{y}\\ where\;s=\sum \sqrt{\Delta x^{2}+\Delta y^{2}}\;and\;s_{i}^{p}=\sum \sqrt{\Delta {x_{i}^{p}}^{2}+\Delta {y_{i}^{p}}^{2}} \end{array}$$

where the arc length *s* is the cumulative sum of the referential path and coefficients α,β,γ, and δ in the cubic spline can be calculated using the boundary conditions of the first derivatives and second derivatives. Thus, all the path candidates $p_{candidate}$ can be interpolated with the cubic spline and can be considered the curvature of the global path. In addition, the curvature κ and heading ψ of the path candidate can be calculated by Equation (4), and the results of path candidates generation are shown in Figure 6.

(4) $$\begin{array}{c} \kappa =|\frac{x^{\prime }y^{\prime \prime }-x^{\prime \prime }y^{\prime }}{{\left({x\prime }^{2}+{y\prime }^{2}\right)}^{\frac{3}{2}}}|\\ \psi =atan(\frac{y^{\prime }}{x^{\prime }}) \end{array}$$

The next step involved selecting the minimum-cost path among the candidates. All the candidates were penalized according to the jerkiness, the proximity from the detected obstacles, and curvature following the proposition in [29]. As for the proximity from the obstacles, there was a large heuristic penalty implying that the obstacle had collided or was located within the value set for the safety distance $d_{collide}$. Here, g(t) is the function of proximity, and $\kappa_{candidate}$ is the function of curvature according to Equation (4). Therefore, the cost function C is defined as:

(5) $$\begin{array}{cc} \; & C=k_{j}J_{t}+k_{c}g\left(t\right)+k_{R}\kappa_{candidate}\\ \; & where\;J\left(p_{candidate}\left(t\right)\right)=\int_{t_{0}}^{t_{1}}{\dddot{p}}_{candidate}^{2}\left(t\right)d\tau \\ \; & g\left(t\right)=\left\{\begin{array}{cc} infinite & \mathrm{if}\;\min_{i}\left|p_{candidate}\left(t\right)-O_{f}\right|<d_{collide}\\ \frac{1}{\min_{i}\left|p_{candidate}\left(t\right)-O_{f}\right|} & \mathrm{else} \end{array}\right.\\ \; & \kappa_{candidate}=\frac{{\ddot{p}}_{candidate}\left(t\right)}{{\left(1+{\dot{p}}_{candidate}{\left(t\right)}^{2}\right)}^{\frac{3}{2}}} \end{array}$$

However, the limited numbers of path candidates generated using the road-model-based approach could not always guarantee a collision-free path. An example is the case where a more complex path generation is required in abnormal environments where various obstacles are detected and should be avoided owing to accidents in the forward multiple lanes. Therefore, if all the generated path candidates had high costs, we generated the path by triggering the graph-based local path planner. The graph-based path planner created a collision-free path using the hybrid A-star algorithm [30] based on the occupancy grid map information generated in the BLOS perception subsystem.

#### 4.2.3. Behavior Planning

**Figure 7:**
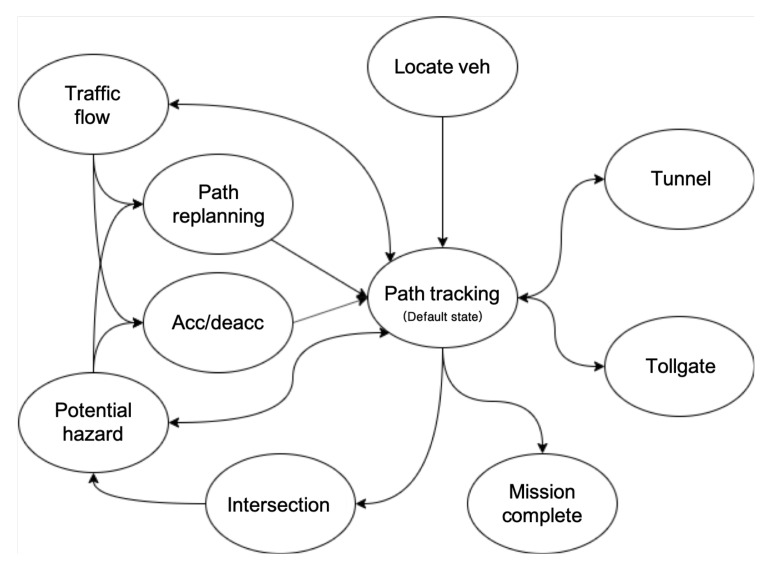
FSM for behavior planning of Eurecar.

The behavior planning subsystem was responsible for planning the state of the vehicle until it reached its destination. As stated earlier [31], using a finite state machine (FSM) for behavior or mission planning makes it possible to prevent the autonomous vehicle from getting stuck before the end of its task. Eurecar’s behavior planning system was designed based on urban driving scenarios and had a total of 11 possible states (Figure 7). The descriptions of each state are as follows:

- Locate veh: This is the initial state of the behavior planner. In this state, the autonomous vehicle locates its ego-position using GPS and IMU information.
- Path tracking: As the default state, this state corresponds to performing longitudinal and lateral control along with the velocity profile while following the global path.
- Potential hazard: This state is invoked when the potential hazard is higher than a certain threshold owing to the sudden appearance of a pedestrian, detection of an accident vehicle ahead, and the like. To decrease the potential hazard, the state is transferred to the acc/deacc state or the path replanning state, which creates a new path for collision avoidance.
- Traffic flow:This state is invoked when receiving a V2X TIM message that conveys the construction site location and accident information ahead or an SPAT message containing the traffic light status and remaining time.
- Acc/deacc: In this state, autonomous vehicles perform acceleration or deceleration.
- Path replanning: This state can be invoked from the potential hazard state and traffic flow states. When called from the Potential hazard state, the path is planned based on the A-star algorithm to avoid collisions with obstacles ahead (Section 4.2.2). Otherwise, when called from the traffic flow state, the route is replanned using the Dijkstra algorithm based on the V2X information (Section 4.2.1).
- Intersection: Using the information received in the V2I message, the autonomous vehicle can pass through the intersection at the optimum speed without unnecessary deceleration. At this time, if another vehicle passing through the intersection is detected by V2V or the sensors, speed planning is performed in response to the surrounding vehicles.
- Tunnel: This state is invoked when passing through a tunnel section with low GPS signal accuracy. In this state, Eurecar switches its lateral controller to RT recognition-based lane keeping, thus not following the waypoint based on the position of the vehicle.
- Tollgate: This state is invoked when the vehicle has to pass through a tollgate.
- Mission complete: This state is reached when all the missions are over and the vehicle stops.

#### 4.2.4. Velocity Planning

The velocity planner aims to set an appropriate speed for autonomous driving to follow a route and is responsible for planning acceleration and deceleration to comply with traffic situations, such as sudden stops due to obstacles during driving. We configured behavior planning to generate the target velocity according to the state of the FSM used in behavior planning. In the Path tracking state, the autonomous vehicle follows the velocity profile created in an offline manner. The velocity profile was generated based on an earlier study [32]. The planner generates velocity profiles that do not violate lateral friction constraints. However, one modification made to the algorithm was that the desired latitudinal acceleration was used instead of the maximum static friction coefficient as follows. The centripetal force equilibrium during rotation with friction constraints can be described using Newton’s law as:

(6) $$F=ma_{lat}=\frac{mv^{2}}{R}<m\mu g$$

which leads to

(7) $$v_{curve}=\sqrt{a_{lat}R}$$

Here, *F* is the centrifugal force, *v* is the longitudinal velocity, $a_{lat}$ is the desired latitudinal acceleration with $a_{lat}<\mu g$, *R* is the turn radius during motion, and μ is the static friction coefficient. If some part of the planned velocity exceeds the longitudinal acceleration limit, the planner replans the dynamically infeasible part so that the command becomes feasible based on the longitudinal acceleration limit $a_{lon}$. The velocity profile generator explained above is illustrated in Algorithm 2.


**Algorithm 2:** Velocity profile generating algorithm based on the turn radius of the given path.

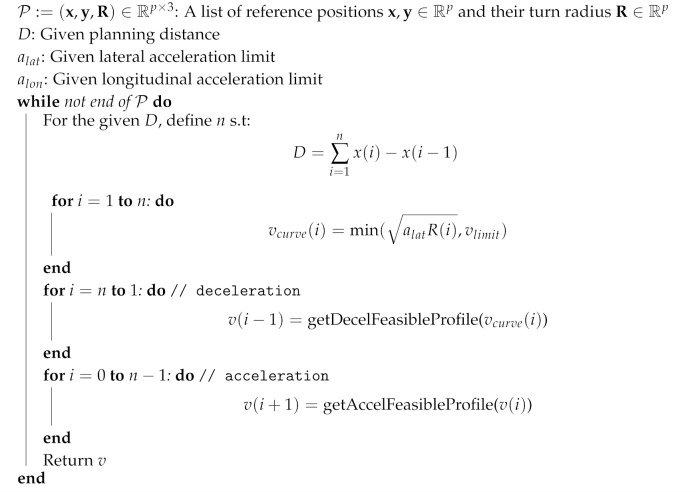




In the traffic flow state, the speed limit is transmitted via V2I (TIM message). Eurecar sets the maximum velocity to the speed limit and stops generating velocity commands above the velocity limits. In the intersection state, using the traffic signal time and state transmitted by V2I (SPAT and MAP messages), the velocity planner plans the target velocity at which autonomous vehicles can cross the intersection without unnecessary acceleration or deceleration. A traffic signal status is represented as $\left[\phi_{red},\phi_{yellow},\phi_{green}\right]\in \phi_{inter}$; the distance to the intersection stop line $d_{stop}$ is calculated to pin the HD map, and the remaining traffic signal time used for velocity planning $\tau_{inter}$ is given as follows:

(8) $$\tau_{inter}=\left\{\begin{array}{cc} \tau_{green} & \mathrm{if}\;\phi_{green}\;\mathrm{and}\;d_{stop}<v_{cur}\tau_{green}+\frac{1}{2}a_{lon}\tau_{green}^{2}\\ \tau_{red}^{max}+\tau_{yellow}^{max}+\tau_{green} & \mathrm{if}\;\phi_{green}\;\mathrm{and}\;d_{stop}>v_{cur}\tau_{green}+\frac{1}{2}a_{lon}\tau_{green}^{2}\\ \tau_{red}^{max}+\tau_{yellow} & \mathrm{if}\;\phi_{yellow}\\ \tau_{red} & \mathrm{if}\;\phi_{red} \end{array}\right.$$

The speed when passing through the intersection without unnecessary deceleration is:

(9) $$v_{inter}=\frac{d_{stop}}{\tau_{inter}}$$

### 4.3. Control

#### 4.3.1. Longitudinal Control

**Figure 8:**
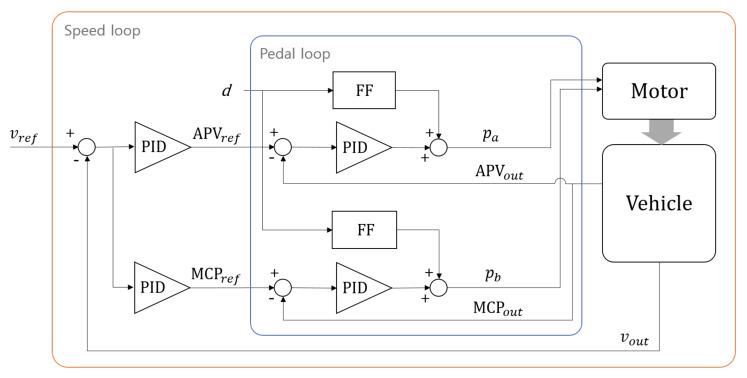
Schematic of longitudinal low-level control. Vehicle speed is controlled in a cascade manner with an outer speed loop and an inner pedal loop.

The computed velocity command explained above is then fed into the low-level controller. It has a cascade feedback form, consisting of a pedal control loop inside of the speed control loop. The current velocity of the vehicle is fed back to the speed loop, giving the pedal command, reference acceleration pedal value (APV), and reference master cylinder pressure (MCP) to the inner loop. Again, the current pedal values, $APV_{out}$ and $MCP_{out}$, are fed back to the pedal loop, giving control commands to the motors that actuate the pedals. All the resistant forces are treated as a unified disturbance *d* and are compensated through feedforward compensation in the pedal loop, as illustrated in Figure 8.

#### 4.3.2. Lateral Control

Among various path following algorithms, the Stanley method [33] was implemented for its simplicity in the latitudinal controller of Eurecar. The modification we made to the standard Stanley method was using the “look-ahead point”. Although the Stanley method is exponentially stable, our experiments indicated that this was not actually true as we observed large initial cross-track errors, typically greater than 1 m. In fact, large initial errors caused overshoots during the transient. One may argue that decreasing the control gain could solve this issue. However, this would not be a good solution because gains need to be large enough to maintain sufficient tracking performance. By implementing the look-ahead point with length $L_{p}$ measured from the center of the rear wheels, the proposed method was equivalent to treating the vehicle as if it had a longer wheelbase. As a result, a more robust response could be achieved.

**Figure 9:**
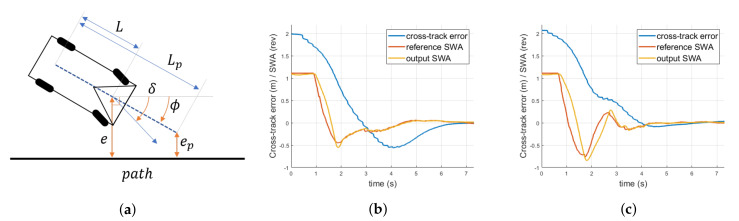
(**a**) Schematic of the kinematic model for the proposed path-following controller. (**b**) Experimental step response of original Stanley method with L = 2.65 m with the gain *k* = 0.5/s and speed *v* = 6 m/s, showing cross-track error in meters and steering wheel angles in revolutions. (**c**) Experimental step response of the proposed method with L = 2.65 m and $L_{p}$ = 5.3 m. Faster convergence with less overshoot was observed using the proposed method.

Analysis of the proposed path following control is presented as follows. From the geometric relations obtained from Figure 9a,

(10) $$e_{p}=e+\left(L_{p}-L\right)\sin\psi$$

where ψ is the heading error (yaw angle) of the vehicle from the closest path. The derivative of the cross-track error and that of the yaw angle are given as

(11) $$\dot{e}=v\sin\left(\psi -\delta \right)$$

and:

(12) $$\dot{\psi }=-\frac{v}{L}\sin\delta$$

where δ is the front wheel angle from the center of the vehicle. Implementing the Stanley method yielded the following control law and its derivatives:

(13) $$\delta =\left\{\begin{array}{cc} \psi +\arctan(\frac{ke_{p}}{v}) & \mathrm{if}\;|\psi +\arctan\left(\frac{ke_{p}}{v}\right)|<\delta_{max}\\ \delta_{max} & \mathrm{if}\;\psi +\arctan\left(\frac{ke_{p}}{v}\right)\geq \delta_{max}\\ -\delta_{max} & \mathrm{if}\;\psi +\arctan\left(\frac{ke_{p}}{v}\right)\leq -\delta_{max} \end{array}\right.$$

(14) $$\dot{\delta }=\left\{\begin{array}{cc} \dot{\psi }+\frac{k}{v}\frac{\dot{e}+\left(L_{p}-L\right)\dot{\psi }\cos\psi }{1+{\left(\frac{k}{v}\left(e+\left(L_{p}-L\right)\sin\psi \right)\right)}^{2}} & \mathrm{if}\;|\delta |<\delta_{max}\\ 0 & \mathrm{if}\;\left|\delta \right|\geq \delta_{max} \end{array}\right.$$

Figure 9b,c depicts the experimental step response of each control law. It was evident that utilizing look-ahead point $L_{p}$ and its cross-track error $e_{p}$ gave a smoother and faster response with considerably lesser overshoot than the original one. Using the look-ahead point, Eurecar could safely maintain stability even in discontinuous conversion during local planning.

## 5. Experimental Results

To verify the performance of the proposed autonomous driving system developed in this study, the system was integrated into a full-scale vehicle and participated in the 2019 AVC. The 2019 AVC took place at K-city, an autonomous driving test bed equipped with V2X infrastructure.

**Figure 10:**
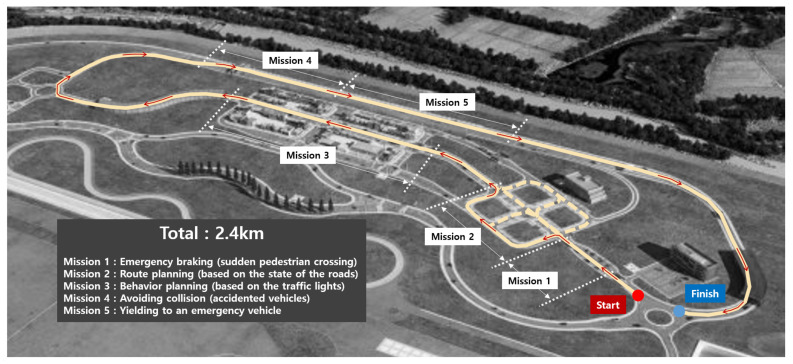
2019 Hyundai AVC route in K-city.

As shown in Figure 10, the travel route was approximately 2.4 km and the red and blue dots indicate the start and finish positions, respectively. There were five missions in the AVC, and each was designed based on various scenarios that could occur in real road situations. The final winner of the AVC was determined based on the sum of the travel time and penalties incurred for each mission. With the proposed V2X-aided autonomous driving system, Eurecar ran at a top speed of approximately 70 km/h and recorded the fastest lap time (3 min and 30 s) among the 12 teams in the final round without mission failure. The final driving video is available at https://youtu.be/BuyF6w7L6JM. In the following sections, we present the results obtained using the integrated autonomous driving system involving significant events.

### 5.1. Mission 1. Emergency Braking Due to a Jaywalker

**Figure 11:**
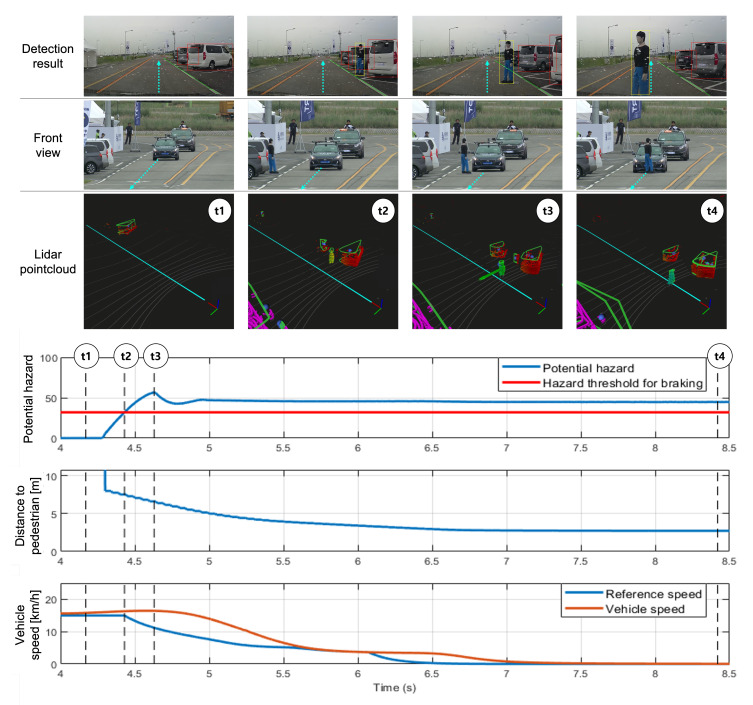
Result of Mission 1. At t1, Eurecar ran along the global path. At time t2 where the potential hazard exceeded a certain threshold, a deceleration command was sent to the low-level controller. The maximum potential hazard due to control delay and vehicle characteristics was at t3. At t4, the pedestrian stopped in the middle of the roadway. Eurecar stopped until the pedestrian crosses.

Mission 1 was considered successful if the vehicle stopped within 3 m, avoiding collision when a jaywalker accidentally jumped on to the road from a random location. This road section consisted of a single lane, and the mission could be restarted after the jaywalker completely moved out of the lane. As the location and velocity information of the pedestrian was not provided through V2X, recognition of the pedestrian was performed with the on-board sensory system and LiDAR sensors. The first and third rows of Figure 11 are the vision-based detection results (considering the neighboring vehicles, pedestrian, and lane conditions) and that based on the three-dimensional information obtained via LiDAR sensors, respectively. Additionally, third-person views are included in the second row, and the path followed by the vehicle is overlaid with dashed arrows.

With the start signal, Eurecar followed the global path (as shown in cyan color) and calculated the potential hazard ahead repeatedly. When there was no obstacle ahead, the potential hazard was initialized to zero. Between t1 and t2, Eurecar detected the jaywalker for the first time, and the potential hazard increased rapidly. At t2, the potential hazard exceeded a preset threshold (we empirically set this threshold to 37), and the vehicle started decelerating. Eventually, Eurecar completely stopped when the pedestrian was located within 3 m of the front wheel.

### 5.2. Mission 2. Detouring the Construction Sites

**Figure 12:**
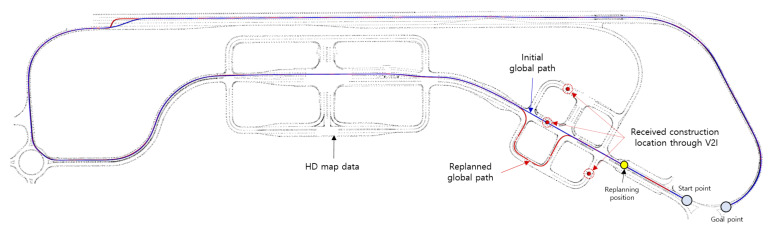
Result of Mission 2. Global locations of the construction sites received via V2I are marked with red dots, whereas blue and red lines indicate the initially provided path and the replanned path that made a detour around the construction sites, respectively.

Mission 2 was conducted in an area where multiple routes could be selected to pass through a specific section. During the competition, it was assumed that construction was in progress along some routes, and this information was transmitted through V2I communication. Moreover, there was a rule that vehicles could not pass through the section with the construction sites. The global location of the construction sites received at the time of the competition was (N: 37.239812, E: 126.773170), (N: 37.240313, E: 126.773916) and (N: 37.240148, E: 126.774420) in GPS coordinates, and this is marked with a red dot in Figure 12. Using the global path planner introduced in Section 4.2.1, Eurecar replanned a global path detouring the construction sites and tracked it without lane invasion. Figure 12 shows the initial global path (blue line) and the trajectory based on the replanned global path (red line).

### 5.3. Mission 3. Complying with Traffic Signals

**Figure 13:**
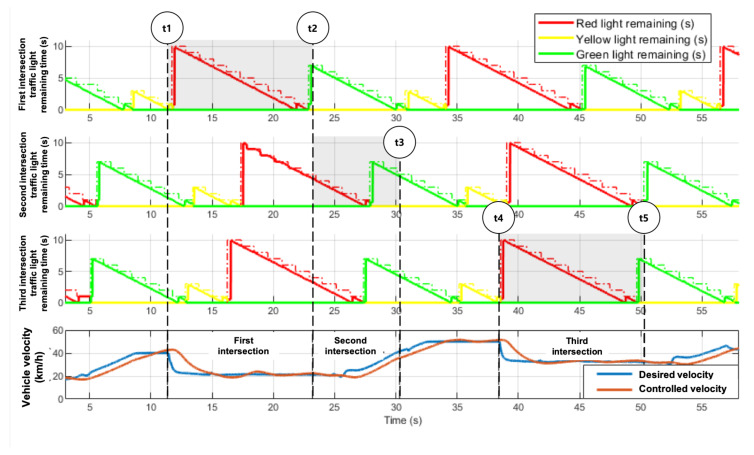
Velocity profile and longitudinal control result in Mission 3. The top three rows depict the traffic light status at each intersection. Dotted and solid lines indicate remaining time received via V2I (1 Hz) and upsampled results (10 Hz) using an internal clock. At t1, the behavior planner changed the state to the intersection state. At t2, Eurecar drove through the first intersection without stopping. Because the remaining time of the red traffic light of the subsequent intersection was short, further deceleration was not required, and acceleration was performed in consideration of the signal state at the last intersection (t3). In the same way (from t1 to t2), the Eurecar passed the last intersection without stopping (from t4 to t5).

Mission 3 took place in an urban environment with consecutive intersections. There were three intersections in total, and the mission involved passing through the intersections while obeying the traffic signals. If the vehicle violated the stop line or did not follow traffic signals, a two minute penalty was added to the total lap time. Within a section 30 m before the first intersection to the last intersection, the state changed to the intersection state according to behavior planning. In this state, to reduce the lap time, mission-specific velocity planning was carried out according to Equation (13) using SPAT and MAP messages. These V2I messages were upsampled from originally 1 Hz to 10 Hz by adding an internal clock for more accurate velocity planning. The top three rows of Figure 13 show the traffic light status of each intersection. The received V2I messages (1 Hz) are represented using dashed lines, and the upsampled data (10 Hz) are denoted using a solid line. The bottom row of Figure 13 shows the desired and controlled velocity.

### 5.4. Mission 4. Collision Avoidance

**Figure 14:**
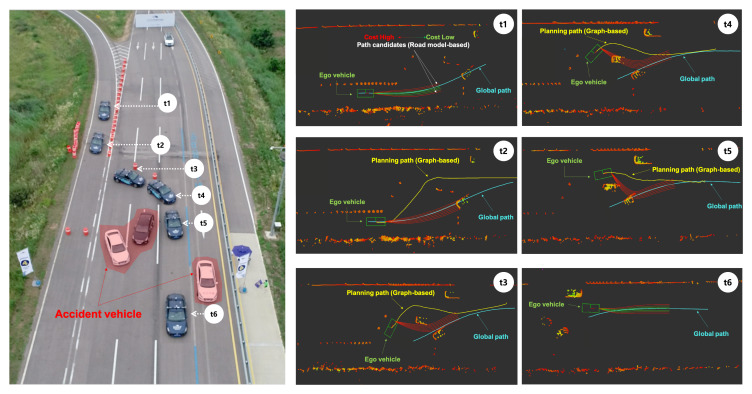
Results of collision avoidance mission. At time t1, Eurecar moved along the minimum-cost path among the candidates around the global path. At time t2, all candidate paths collided and were not feasible, thus leading to the creation of a collision avoidance path with the graph-based approach. From t3 to t5, Eurecar tracked the path generated by the graph-based approach, and at t6, Eurecar merged to the initial global path developed through the road-model-based approach.

In Mission 4, it was assumed that an accident had occurred ahead and the autonomous vehicle must pass through the area without any collision. The driving environment consisted of five lanes, as shown in Figure 14, with obstacles including accident cars and cones. As obstacle placement at the accident site was not done in advance, it was essential to create a collision avoidance path in real time based on locally sensed data.

In Figure 14, the drone view and logged data of Eurecar performing collision avoidance are displayed in chronological order. Before t2, Eurecar followed the cost-minimum path among the candidate paths created around the global path (cyan color). At t2, Eurecar first determined that the global path could not include an accident vehicle and created a path (yellow line) to avoid this using the hybrid A-star algorithm. While traveling along the replanned path (from t3 to t5), Eurecar generated the path candidates to merge into the global path in parallel. At t6, a path candidate that could participate in the global path and join the global path safely was selected. Eurecar followed the cost-minimum path among the candidate paths created around the global path. When all candidate paths collided or were not feasible, the hybrid A-star algorithm created collision-free paths.

### 5.5. Mission 5. Yielding the Ego-Lane to an Emergency Vehicle

**Figure 15:**
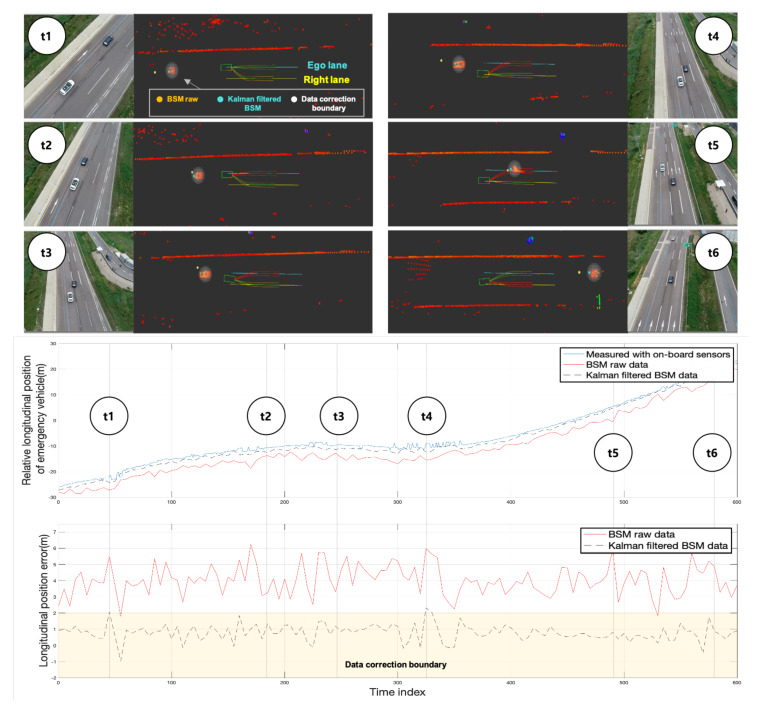
Results of Mission 5. At time t1, Eurecar received the basic safety message (BSM) of the emergency vehicle approaching from behind while driving along the ego-lane. At times t2 and t3, the emergency vehicle approached within 10m, and a lane change to the right was performed by arbitrarily increasing the cost of path candidates generated around the ego-lane to yield lanes. At times t4 and t5, the emergency vehicle had not yet passed, and Eurecar ran along the changed lane. At time t6, the Eurecar changed the lane to the left to return to the original lane.

Mission 5 was conducted after passing through the accident zone. This mission involved yielding an ego-lane within 5 s when an emergency vehicle approached within 10 m from the rear. The emergency vehicle transmitted information including its location, velocity, and vehicle type via V2V, as a BSM. The location of the emergency vehicle was estimated by the Kalman filter using the speed and orientation information contained in the BSM via the BLOS perception algorithm. Additionally, the estimated position using BSM was compared with the data correction boundary on the centroid of the clustered LiDAR point clouds, and data correction was performed. When the rear vehicle approached within 10 m, the minimum-cost path was selected among the road-model-based candidate paths for changing lanes and moving to the right lane. Figure 15 shows the recognition results of the rear vehicle obtained through the BLOS perception subsystem and road-model-based path candidates for lane change, with the rear emergency vehicle approaching, at different times during the mission.

## 6. Conclusions

In this study, we developed a V2X communication-aided autonomous driving system and integrated it into a full-scale autonomous vehicle platform to verify its performance. The proposed system was designed such that V2X information played a complementary role in perception, planning, and control considering extant research pertaining to autonomous driving systems. The BLOS perception system was developed through data fusion between local perception using on-board sensors and communicated perception using V2X for road safety, especially in occlusion environments, such as those at intersections. In addition, by utilizing various types of traffic information provided through V2I for planning, an extended planning system for autonomous vehicles was developed to generate routes and comply with traffic regulations based on RT traffic information.

Using the proposed system, vehicles can be made fully autonomous in K-city built using V2X communication infrastructure. The authors participated in the 2019 Hyundai AVC, wherein their autonomous vehicle equipped with the proposed driving system successfully completed all assigned missions based on several scenarios, including those pertaining to emergency braking caused by a jaywalker, detours around construction sites, complying with traffic rules, collision avoidance, and yielding the ego-lane for emergency vehicles.

The potential of the proposed system was confirmed based on scenarios wherein the V2X communication technology was used for autonomous driving. However, the proposed system can be further improved to tackle several real-world traffic scenarios. For example, in situations involving a non-signalized intersection, the current system warns of an imminent collision en route and decelerates the vehicle rather than perceiving the intentions of other vehicles. Additionally, further experiments need to be performed considering a wider variety of traffic entities, such as bicycles and cheating vehicles. The authors plan to expand the scope of this research, thereby improving the proposed system and making the concept of fully autonomous vehicles a reality in the near future.

## Figures and Tables

**Table 1:** Strengths and weaknesses of local and communication perceptions.

	Local Perception (With On-Board Sensors)	Communicated Perception (With V2X Communication)
Strength	-Fast object detection rate (>20 Hz) -Precise object localization within short range (<50 m)	-Unlimited line-of-sight -Intensive information
Weakness	-Limited line-of-sight -Easily affected by environmental conditions -Expensive sensors	-Low update rate (<10 Hz) and traffic loss -Not very accurate (position, velocity, etc.)
